# Parental Stress, Anxiety and Depression Symptoms Associated with Self-Efficacy in Paediatric Type 1 Diabetes: A Literature Review

**DOI:** 10.3390/ijerph18010152

**Published:** 2020-12-28

**Authors:** Giulia Bassi, Elisa Mancinelli, Daniela Di Riso, Silvia Salcuni

**Affiliations:** 1 Department of Developmental Psychology and Socialization, University of Padova, 35132 Padova, Italy; elisa.mancinelli@studenti.unipd.it (E.M.); daniela.diriso@unipd.it (D.D.R.); silvia.salcuni@unipd.it (S.S.); 2 Fondazione Bruno Kessler, 38123 Trento, Italy

**Keywords:** parental self-efficacy, parental stress, parental diabetes-specific distress, paediatric Type 1 diabetes mellitus, review

## Abstract

*Background*: Parents play a significant role in the management and monitoring of their children’s Type 1 diabetes mellitus (T1DM), being considered a family disease. The current review intends to investigate parental stress, depression and anxiety symptoms associated with self-efficacy referred to paediatric diabetes management. *Method*: A literature review was carried out using PsycINFO, Web of Science and PubMed where the following terms were considered: diabetes mellitus, paediatric, parent-child relationship, self-efficacy, parenting stress, perceived stress, stress, depression, anxiety. Standing a defined list of inclusion and exclusion criteria, a total of 33 papers were finally included. *Results*: Findings have shown that parents experience relatively high levels of anxiety, depression and stress symptoms related to managing a child with T1DM and are associated with parental self-efficacy. Parental stress predicts a worsening in the control of HbA1c levels, while parental diabetes-specific distress predicts an increase in children depression symptoms. High parental self-efficacy associates with better monitoring, allowing better adherence and more balanced HbA1c levels in the children. *Conclusions*: Interventions aimed at fostering social support, improving diabetes management, and decreasing perceived stress, might alleviate parents’ psychological symptoms by focusing on increasing their self-efficacy. Digital interventions might also represent valuable solutions to support parents in the management of paediatric diabetes not presented and substantiated in the main text and should not exaggerate the main conclusions.

## 1. Introduction

Type 1 diabetes mellitus (T1DM) is one of the most common paediatric chronic illnesses, with a prevalence of 1 in every 400 youth [1] and preceded only by asthma and epilepsy [2]. Paediatric T1DM involves many life-challenges, as children and adolescents are forced to follow an inflexible regimen. Insulin injections, physical activity and a healthy dieting to avoid episodes of extremely high or low levels of hemoglobin A1c (HbA1c), both of which can put their life at risk [3]. The ongoing management of T1DM can also be extremely challenging for parents, as requiring family support for both the physical and psychological care of the children [4]. Parents play a significant role in the management and in the monitoring of their children and adolescents’ T1DM regimen-related tasks as continuously aimed at maintaining balanced their glycaemic levels. Indeed, some studies, line with the transactional model of parent-child interaction, in which the child’s illness affects the family at different levels, then affecting back on the health of the child with diabetes, suggest that it represents a family disease [5,6]. Therefore, since parents have to adapt their approach to managing their children’s chronic disease, they may experience several psychological symptoms. Anxiety, stress and/or depression related to paediatric diabetes, which in turn, influence the glycemic control of their children’s diabetes [7,8]. More specifically, anxiety represents an emotion constituted of feelings of worrying thoughts, tension, and physical modifications (e.g., the increase of blood pressure) [9]; stress is a physiological or psychological response to internal or external stressors [9] while the perceived stress comprises feelings or thoughts regarding the general stressfulness of the individuals’ life and their capacity to handle this stress [10]. Parental stress, instead, is defined as the parental perception of an imbalance between the requests of parenting and accessible sources [11]. Lastly, depression is characterized by a negative affective state, which ranges from unhappiness to an extreme feeling of sadness, despondency, and pessimism, as well as changes in physical, cognitive, and social aspects [9], which altogether interferes with everyday life. According to the literature, parental responses referred to stress, anxiety and depression are common following their children diabetes diagnosis, especially regarding thoughts of possible acute complications [12]. The American Diabetes Association (ADA), indeed, suggests adopting a family-centred approach [13] so that parental stress could be addressed by increasing parents’ self-efficacy, which represents the self-confidence related to feeling competent in performing a specific task, and thus relevant as predictive of healthy behaviours [14]. Studies on self-efficacy have suggested that it is an important construct for disease management within the health care setting [15]. As regards paediatric diabetes, parents’ self-efficacy seems central for their well-being related to paediatric diabetes management, as it positively affects both parents and their children [16]. Moreover, parental self-efficacy allows them to overcome the stressors and challenges related to diabetes, thus facilitating better management of the children T1DM [16]. Indeed, parents with a satisfactorily high self-efficacy can use it to buffer the often-reported high levels of stress, encompassing a disease such as T1DM [16]. Therefore, parents’ perception of their self-efficacy seems to play a significant role and impact on their psychological well-being, which could, in turn, influence the management of their children chronic disease. Up until now, little has been reviewed regarding parental psychological symptoms related to the management of paediatric T1DM, especially regarding the role of parental self-efficacy [17]. Thus, the purpose of the present literature review is to define a comprehensive outline of the past literature, especially focusing on anxiety, depression and stress symptoms related to parental self-efficacy and referred to the management of children and adolescents’ T1DM, with self-efficacy conceived as a protective factor towards the whole family well-being. 

## 2. Materials and Methods 

### Search Strategy and Inclusion and Exclusion Criteria

**Figure 1:**
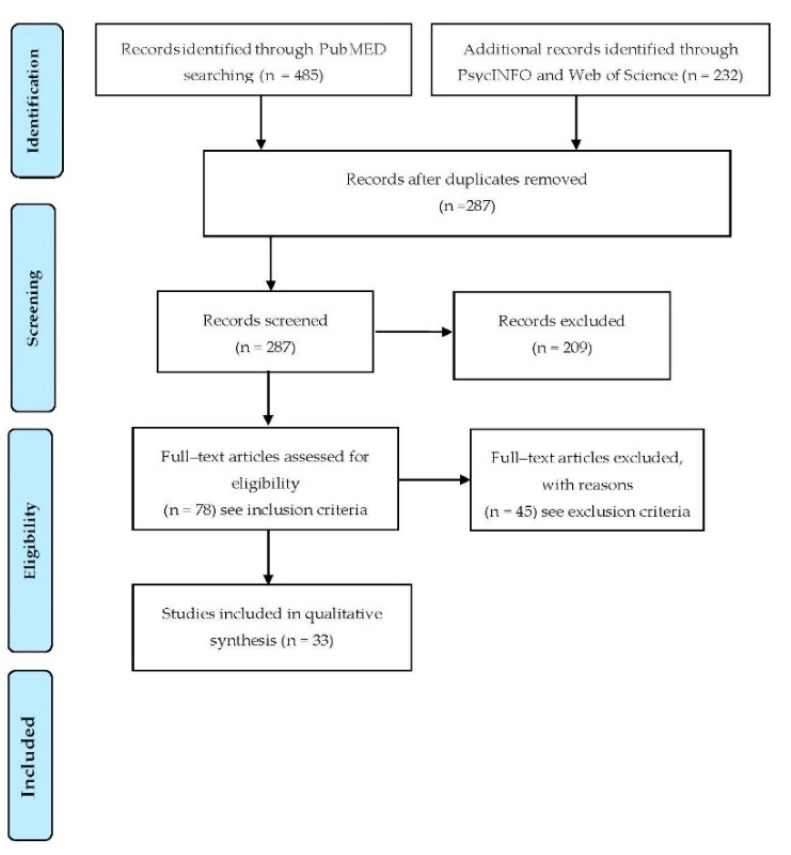
Preferred Reported Items for Systematic Reviews and Meta–analyses (PRISMA) chart summarizing the selection process.

Following the PRISMA Group workflow (Figure 1), a literature review has been conducted through the PubMed, Web of Science and PsycINFO academic databases, where the following keywords and MESH terms were considered, as well as their derivatives, during the search: diabetes mellitus, paediatric, Type 1 diabetes mellitus, children, adolescents, parent-child relationship, parental management, self-efficacy, parenting stress, depression, anxiety, perceived stress, stress. To be included in the review process studies should consider: (i) one or both parents involved in the management of their children or adolescents’ T1DM, and at least one of these two conditions: (ii) measures of parents’ psychological symptoms associated to stress, anxiety and/or depression symptoms, (iii) the role of parental self-efficacy. Studies should also (iv) be fully written in English. (v) Review articles and dissertations meeting the above inclusion criteria were also considered. Studies that met any of the following criteria were excluded from the study: (i) full-text was not available; (ii) focus on adults with T1DM or Type 2 diabetes mellitus (T2DM); (iii) focus only on the psychological well-being of children or adolescents with T1DM; (iv) consideration of children at risk for T1DM with no effective diagnosis of diabetes; (v) parents presenting post-traumatic stress disorders (PTSD) related to their children’s T1DM; (vi) preceding diagnosis of postpartum depression; (vii) consideration of children or adolescents with other chronic diseases; (viii) the presence of children and adolescents with clinical disorders in comorbidity; (ix) the presence of parental antidepressant treatment.

## 3. Results

The literature search has highlighted a total of 33 studies (one review and 32 research articles), which consider parents of children and adolescents with T1DM, aged between 1 to 18 years (Table 1).

### 3.1. Parental Depression and Anxiety Symptoms 

Several studies reported that parents of children with T1DM experience higher levels of depression and anxiety symptoms, relevant as they associate with difficulty coping with the children’s T1DM [18,19,20,21]. Nonetheless, findings are not univocal, with some reporting a greater prevalence of depression symptoms [22,23] while others of anxiety symptoms [19,24] in both mothers and fathers. A recent study highlighted that most of the parents experience paediatric-specific distress following their children’s diagnosis of T1DM, then worsened by depression symptoms [25]. Indeed, the authors found that parents with depression symptoms show higher levels of daily T1DM-specific distress compared to parents without depression symptoms at baseline [25]. Moreover, parents reporting such symptoms at baseline also show a reduced improvement in T1DM-specific distress assessed at six- and twelve-month follow-up [25]. A 2012 systematic review reports a prevalence of 33.5% regarding parents’ distress at the time of diagnosis and 19% 1 to 4 years after the diagnosis with a prevalence of parental psychological distress ranging from 10% to 74% [21]. Indeed, other evidence coherently observed that 74% of parents met mild depression and 61% met criteria for clinically significant depression [20]. A study also showed that 13% of parents present depression symptoms within a mild to moderate [26]. Along the same line, another study report that 33% of parents showed elevated symptoms of depression; the best predictors, assessed through hierarchical multiple regression analysis and logistic regression, were the caregivers low education levels, higher family stress, the children’s older age and low levels of HbA1c [27]. For instance, parental depression had a significant indirect effect on children metabolic control through parental monitoring [28]. Hansen and colleagues [18] report that 55% of mothers and 22% of fathers met the cut-off for an anxiety disorder, while for depression disorders the 26% of mothers and 19% of fathers fell above the cut-off. Similarly, in a 2013 study, 29.8% of parents scored within the moderate range or above the cut-off for severe anxiety disorders, while there was no significant difference in depression symptoms compared to the control group [19]. In another study, 59% of parents reported clinically significant levels of anxiety at the time of their children diabetes diagnosis [20]. Data, referred to parents of adolescents with T1DM, highlight that the relationship between the caregivers’ psychological distress and the HbA1c levels is stronger for depression symptoms than anxiety symptoms [23]. Furthermore, data also show that parents’ depression symptoms have a positive and significant indirect effect on their children depression symptoms when parental involvement in their children with T1DM is considered [28].

### 3.2. Parental Stress and Parenting Stress

Helgeson and colleagues [5] distinguished between general stress, related to the areas of finances, marriage, and parenting, and parental stress or diabetes-specific stress referring to the stress experienced when caring for a child with diabetes. Indeed, general parental stress predicts an increase in parents’ depression symptoms, which significantly associates with more children depression symptoms and lower glycemic control. Furthermore, high levels of general stress represent one of the risk factors for the onset of depression symptoms in parents caring for children with T1DM [29]. Parents who report their children’s behaviour as more problematic, showed greater difficulties associated to paediatric parenting stress and increased child-reported critical parenting behaviours [18,30]. Moreover, maternal illness related stress associates with the children’s mental state [31]. Considering the differences referred to children’s age, parents of children (Mage = 10.8 years) report, overall, less paediatric parenting stress compared to parents of preadolescents (Mage = 12.9 years) [26]. Another study showed that parents of children aged between 2 to 7 years present higher levels of paediatric parenting stress frequency referred to the management of children with diabetes while paediatric parenting stress difficulties was associated with greater parental depression symptoms [25]. Regression analysis showed that parental depression symptoms explain 58% of the variance of paediatric parenting stress frequency and 68% of the variance of paediatric parenting stress difficulties [25]. Moreover, a study evaluating the validity of the Paediatric Inventory for Parents (PIP; a self-report aimed at assessing stress levels in parents with a child presenting a chronic illness) showed the positive predictive role of three domains, namely communication frequency (i.e., talking about the child illness), role function frequency (i.e., issues referred to the caregiver role as a parent, partner and person with their own need) and emotional problems frequency (i.e., negative emotionality referred to the child illness) [32]. Differently, parents’ age appeared as negatively predicting anxiety [33]. Considering gender differences should be noted how studies present contrasting results since on the one hand they report that mothers show higher levels of paediatric parenting stress than fathers [33]. In contrast, a recent pilot study found that mothers and fathers seem to report comparable stress levels related to parenting a child with diabetes [34]. Of particular note is a study in which the sleep issues of children with T1DM has been investigated in association with family functioning and parental psychological symptoms [35]. This study showed that children’s sleep issues and behavioural insomnia associates with increased parental stress, anxiety, and depression symptoms, influencing the intensive insulin regimen. Thus, the management of paediatric diabetes also impacts the children sleep and emotional functioning, while also affecting parents’ sleep as the 79% of them reported on sleep disruption associated to diabetes-management [35]. Therefore, paediatric parenting stress is common in parents of children and adolescents with T1DM, resulting in adverse outcomes for children’s health [20,23,26,36], although further studies are needed to allow an in-depth comprehension of the considered symptoms and the role of parenting stress associated with the management of paediatric T1DM.

### 3.3. The Role of Parental Self-Efficacy Related to Anxiety, Stress, and Depression Symptoms

Parental stress leads to negative consequences for diabetes management as parents perceive T1DM as a complex diagnosis, which contributes to family disruption and leads to anxiety and depression symptoms [21,24]. Evidence underline a constellation of interrelated factors comprising symptoms of stress, anxiety and depression specifically related to the management of the children diabetes [21]. As such, the univocal construct of parental diabetes-specific distress. Indeed, high parental stress, anxiety and depression symptoms, altogether contribute to lowering parental self-efficacy in the context of paediatric diabetes care [20,37]. Further, findings reported parental distress or parental diabetes-specific distress indirectly relates to parents’ diabetes monitoring via self-efficacy [22]. Multivariate analyses showed that parental diabetes-specific stress frequency and difficulties associates with parents psychological and behavioural functioning refers to the perception of greater responsibility and lower self-efficacy related to diabetes management [37]. Low self-efficacy was also related to anxiety and depression symptoms yet positively associated with parents’ younger age. Other authors, indeed, suggest that lower distress in caregivers indirectly relates to better diabetes monitoring through higher parental self-efficacy, in turn associating with their children better adherence and HbA1c levels [22]. Of particular note is that parents’ younger age brings higher parental stress, yet neither to anxiety nor depression symptoms [38]. Moreover, no gender differences were observed for stress difficulty or self-efficacy [38]. Findings further suggest that parental negative affectivity might also negatively influence adolescents’ self-efficacy. Indeed, high parental self-efficacy relates both to diabetes management and to the capacity to develop or reinforce diabetes management related skills to their children, which could positively influence children’s self-efficacy regarding their diabetes self-management [38]. 

### 3.4. Mothers Involvement in the Management of Children with T1DM

Past literature mainly focused on maternal depression symptoms related to parenting children with T1DM. Specifically, after their children diagnosis, mothers report greater depression symptoms than fathers [20]. Maternal depression, related to the burden of paediatric diabetes management, results as the sole significant predictor of adolescents’ glycemic control [23]. Indeed, it was reported that at baseline is associated with reduced maternal diabetes management, while predicting low glycaemic control at three-month follow-up [23]. Moreover, maternal depression symptoms also negatively associated with children’s quality of life, the perception of their coping strategies, and family functioning [39]. Differently, others reported that greater maternal depression did not associate with glycaemic control, yet their adolescents and children were twice as likely to have an emergency room visit and three times more likely to be hospitalized [40]. Wiebe and colleagues [41] observed that the impact of maternal involvement varies as a function of the level of maternal depression symptoms and on the children’s age, with greater caregivers’ depression symptoms leading to higher levels of children and adolescents’ depression symptoms [42,43]. Coherently, in a sample of mothers, 49% of them were above the clinical cut-off for mild depression symptoms, and 25% were above the cut-off for moderate depression symptoms while 26% were above the clinical cut-off for paediatric diabetes distress. As such, mothers’ paediatric diabetes distress -broadly conceptualized as the sum of anxiety, depression and stress symptoms associated to having a child with diabetes- is strongly related to maternal depression symptoms and adolescents’ HbA1c levels [43] although others report that maternal depression and anxiety do not associate with the children metabolic control [39]. Studies considering mothers of adolescents with T1DM show percentages ranging between 18% [44] and 26% [45] for mothers scoring above the clinical cut-off for depression and between 12.9% [45] and 13% [44] for those that have scored above the clinical cut-off for anxiety. Furthermore, highly trait-anxious mothers report holding more responsibility for diabetes management perceiving their adolescents as having less diabetes management-related capacities and as such these adolescents show stronger beliefs of their mothers’ greater control over their diabetes management felt as overprotective [46]. Maternal trait-anxiety was also associated with higher HbA1c levels and greater absenteeism in preadolescents [46] while being correlated to a reduced motivation towards diabetes self-care in older adolescents [46]. Moreover, maternal stress associates to both internalizing and externalizing symptoms in children with T1DM [42] and maternal anxiety and depression symptoms associates with a decreased involvement/warmth towards their children [28]. Contradictorily, others showed that maternal involvement expectancies were related to more monitoring and less conflict, although it does not predict maternal depression symptoms [47]. Nevertheless, the greater consideration of mothers throughout the paediatric diabetes literature could be due to reports of them being more preoccupied with the internal suffering of their children with T1DM, while also showing greater feelings of sadness and anxiety compared to fathers [34]. Maternal depression and anxiety symptoms thus seem strongly interrelated, with depression resulting as the greatest predictor of adverse outcomes on children diabetes than anxiety [23,31]. 

### 3.5. Fathers Involvement in the Management of Children with T1DM

As regards fathers’ involvement in their children’s chronic illness, few studies have addressed this research question. Yet, fathers’ involvement in diabetes care seems to associate with higher parental stress, depression, and anxiety symptoms [18]. Fathers’ paediatric parenting stress positively associates with state-anxiety, although they report greater hope and self-efficacy [48], thus suggesting that fathers may differently experience parenting stress compared to mothers. A recent study showed that poor metabolic control, mediated by the fathers’ perception of their children’s behavioural problems, influences on father-and-child’s dysfunctional interaction as associated to parenting stress [34]. These different experiences may have implications for their level of anxiety and depression and the children’s behaviour [48]. Fathers of adolescents with T1DM report greater parenting stress [36], yet it was observed that decreasing paediatric parenting stress by increasing the perceived social support prompts a decrease in depression and anxiety symptoms [35]. The study showed that paediatric parenting stress explained 25% of the variance in the depression symptoms variance and 18% of their HbA1c levels [36]. Moreover, fathers report relatively high self-efficacy and hope concerning their children with T1DM, although their stress showed no association with the children’s glycemic control [48]. More specifically, fathers’ beliefs and perception of efficacy relate to adolescent’s self-efficacy [49]. Nevertheless, finding of fathers’ psychological symptoms and their involvement in managing children with diabetes need further explorations.

## 4. Discussion

The present paper was to review past literature regarding the impact of children and adolescents’ T1DM on parents’ well-being. The variables considered regarding paediatric diabetes management were parental self-efficacy, parental stress, anxiety, and depression symptoms. In line with the transactional model of parent-child interaction, in which both parties bi-directionally influence each other, parents’ management of their children diabetes and the potentially associated family conflicts, indeed, show consequences upon children’s physical and mental health [5]. Such interactional perspective is particularly important for clinical interventions, as psychological and behavioural variables, directly and indirectly, parents’ and children’s well-being. For this reason, the understanding of how parents are affected by their children diabetes cannot be neglected [5], especially considering parental self-efficacy as a protective factor towards the whole family well-being. 

Parents of children with T1DM seem to be at greater risk for mental health issues [33]. Indeed, studies report parents to show higher levels of depressive as opposed to anxious symptoms compared to the general population [5,27,31] while others observed mothers, in particular, to be often above the clinical cut-off for both depression and anxiety symptoms [22,27]. Moreover, trait-anxious mothers appear as particularly over-protective toward their adolescents and children with T1DM, as they perceived them to be less capable in dealing with their disease [45]. Maternal trait-anxiety was also associated with preadolescents higher HbA1c levels and internalizing problems [45]. Contrarily, fathers tend to experience more state-anxiety [48], yet not much knowledge is present about fathers’ depression symptoms in the context of paediatric T1DM. Furthermore, maternal depression and parental stress were also strongly associated with subsequent negative effects on children’s self-management and lower glycemic control [28,36]. This is not surprising considering the relation between maternal depression symptoms and reduced child-centred parenting behaviours [44], as well as a reduced parental monitoring, thus resulting in low metabolic control [45]. Moreover, high parental self-efficacy permits better monitoring, which in turn allows better adherence and better HbA1c levels of their children by buffering the impact of parents’ diabetes specific distress upon their psychological well-being. Indeed, parental stress, depression, and anxiety symptoms associate with lower parental self-efficacy [20]. As such, implications for clinical interventions should be thoughtfully considered and addressed to improve parental self-efficacy. It is relevant for better managing children’s chronic disease, especially conceptualizing diabetes management within the transactional model of child-parents interaction [5]. Indeed, high family general stress represents one of the risk factors for developing depression symptoms in caregivers of children with T1DM [29], with parental diabetes-specific distress negatively impacting on family cohesion [21,24] and vice versa [24]. Parental anxiety and depression symptoms and diabetes-specific stress may also contribute to the worsening of parental functioning, impacting diabetes management [5,21]. For instance, a five-year study highlighted that parental general stress predicted a decrease in HbA1c levels, while parental diabetes-specific stress predicted an increase in children depression symptoms and better diabetes management [5], which could be due to a greater fastidiousness in dealing with the tasks imposed by the disease. On the same line, maternal symptoms of anxiety and depression are related to reduced child-centred parenting, with anxiety further increasing hostility toward the child, thus jeopardizing mothers’ capacity for disease management while lowering their child’s quality of life. Besides, other studies showed that parental distress related to paediatric diabetes management and comprising only depressive and anxiety symptoms negatively correlates with the HbA1c of their adolescent and children with T1DM [21,23]. Another relevant aspect is that parents need to adapt their behaviours and parenting to the changing necessities as associated to social, emotional, and physical changes. This is relevant for parents’ capacity to manage their children diabetes and to favour adolescents’ capacity to self-manage their disease, which is particularly low during adolescence [23]. Based on these observations, we hypothesized that adolescents’ lower diabetes self-management might be associated with their level of self-efficacy, which could, in turn, be influenced by parental self-efficacy towards diabetes management. For example, results suggest that parental negative affectivity may negatively affect adolescents’ efficacy and self-efficacy [49]. Keeping this in mind, understanding how children’s age and the specific necessities associates with a particular developmental period needs to be further explored, allowing to better tailor clinical care planning. Lastly, it is worth of note that parents can experience other types of stress, which can add up to the management of their children with T1DM. Indeed, parents who showed worries about their financial incomes and their marriage present limited time to support and assist their children with a chronic disease [5]. More specifically, a lower social economic status (SES) contributes to inadequate adherence to the clinical recommendations and to higher parental distress [22], in particular, in parents of younger children and of a non-Caucasian race [37]. Moreover, higher SES was related to better glycaemic control [22] and a lower SES was shown to be a significant predictor of higher maternal anxiety symptoms [39]. Similarly, parents of children with T1DM who show lower education levels were more prone to display depressive symptoms [27]. In this regard, maternal depression was negatively associated with family cohesion [29]; indeed, higher levels of family cohesion were related to a better quality of life and lower levels of parental stress, anxiety and depression symptoms [19]. However, single, or divorced mothers reported to use more maladaptive coping skills compared to married or accompanied mothers [44]. 

## 5. Conclusions

Specific support should be necessary for parents, providing them with tools to face the stress and stressors imposed by a disease like T1DM. To support parents could, indeed, be highly beneficial, both for them and their children since parental depression, anxiety and stress symptoms are strongly interrelated in the management of paediatric diabetes. Moreover, digital solutions can come in hand to facilitate the provision of such interventions. For instance, two studies provided phone-based interventions, which allowed parents to improve their diabetes management capacities [50,51]. A recent eHealth intervention showed improvements in parenting stress in parents of adolescents with T1DM [52]. These interventions based on digital solutions and directed to parents with children with diabetes seem to be enormously appreciated [50,51,52]. Overall, these findings suggest that fathers and mothers react differently to their children and adolescents’ chronic disease and its management. Their perceptions of involvement differently associate with children’s glycemic control and regimen adherence.

## 6. Limitations and Future Works of the Current Review

A limitation in the review is the absence of the consideration of post-traumatic stress disorder (PTSD) and articles referring to it and its symptoms. Articles considering this specific disorder do not specify the criteria parents would be expected to meet, nor if it were present prior or developed due to children or adolescents’ T1DM diagnosis. As such, the present review and future research should identify if such disorder or only some of its specific symptoms are associated to the psychological distress and the parental diabetes-specific stress experienced by parents after their children diagnosis of T1DM. Future studies should examine the hereby-analyzed variables and those mentioned above to evaluate how they translate in a clinical setting. Furthermore, studies should investigate the terms parental specific distress and parental specific stress to allow a more cohesive comprehension on the matter. Future works should consider additional aspects, such as the number of children that parents have to take care of, the differences in the parental management of boys and girls with T1DM, and the role of the potential age differences of the children and adolescents with T1DM on their parents psychological variables and diabetes-management, as the transition between childhood and adolescence poses different challenges for both parents and their child. Moreover, additional works are needed to evaluate the effect of the parental management of children with T1DM as well as their level of depression over time, by considering longitudinal studies. As such, a meta-analysis is needed to quantitatively evaluate the association between the variables considered in the current review as well as the efficacy of relevant treatments on parents with children with T1DM. Lastly, it is worth of note that future studies should consider whether parents show symptoms of depression before their children’s diagnosis, and thus analyzing the relationship between parental depression before their children’s diagnosis and parental management of children with T1DM.

## Figures and Tables

**Table 1:** Main characteristics of the 33 articles reviewed.

Author/Year	Sample (N); Gender (N or %)	Parental Symptoms in Paediatric Diabetes Management	Main Results	Measurement Tools (Considered Cut-Off)
[18] *	*n* = 125 parents (82 mothers and 43 fathers) of children (age ranged between 7–14 years) with T1DM comprising a total of *n* = 89 families.	Stress; depression; anxiety; parents diabetes management	55% of mothers and 22% of fathers met the cut-off for anxiety disorder, while for depression disorder 26% of mothers and 19% of fathers fell above the cut-off. Maternal perceptions of fathers’ helpfulness associates with decreased maternal depression symptoms and a better child regimen adherence Paternal perception of their own amount of involvement was related to increased paediatric parenting stress and anxiety. Their own perception of helpfulness was associated with poorer glycaemic control	Pediatric Inventory for Parents (PIP): self-report assessing parents’ parenting stress specific to caring for a child with a chronic disease. It assesses both the Difficulty and Frequency of the stress-eliciting situations. Hospital Anxiety and Depression Scale (HADS): self-report assessing anxiety and depression symptoms aimed at the general population (clinical cut-off: 8). Self-Care Inventory (SCI): self-report assessing parents’ adherence to diabetes regiment in their children with T1DM.
[19] *	*n* = 104 of parents (90.4% mothers) of children with T1DM (age ranged of 8 to 18 years); *n* = 142 parents (95.8% mothers) of healthy children (control group) (age ranged of 8 to 18 years).	Parental stress; anxiety; depression	29.8% of parents scored within the moderate range or above the severe cut-off for anxiety disorders, while there was no significant difference in depression symptoms compared to the control group	Hospital Anxiety and Depression Scale (HADS): self-report assessing anxiety and depression symptoms aimed at the general population (symptoms severity: “normal” 0–7; “mild” 8–10; “moderate” 11–14; “severe” 15–21). Parenting Stress Index-Short Form (PSI-SF), Parental Distress subscale: self-report assessing parents distress referred to caring and rearing a child.
[20] *	*n* = 102 parents (62.61% mothers) of children (mean age = 9.7 years) with T1DM.	Anxiety; depression; parental stress; self-efficacy	Parents experienced especially high number of depression symptoms. 74% of parents met criteria for mild depression and 61% of the total sample met criteria for clinically significant depression. Parental clinical depression and anxiety were associated with paediatric parenting stress, and with lower self-efficacy for diabetes care.	State-Trait Anxiety Inventory (STAI): self-report assessing state and trait anxiety symptoms (clinical total score cut-off: 40). Center for Epidemiologic Studies Depression (CESD): self-report assessing depression symptoms (clinical total score cut-off: 22). Pediatric Inventory for Parents (PIP): self-report assessing parents’ parenting stress specific to caring for a child with a chronic disease. It assesses both the Difficulty and Frequency of the stress-eliciting situations. Self-Efficacy for Diabetes scale (SED): self-report adopted to assess parent perceived capacity (self-efficacy) to manage their children diabetes.
[21] ****	*n* = 34 articles included (systematic mixed–studies review) regarding parents, children and adolescents with T1DM.	Anxiety; depression; parenting stress; parental distress; parents diabetes management	Parental psychological distress ranged between 10% to 74% after the children’s diagnosis. Parental psychological distress also had negative effects on diabetes management. Themes of the qualitative synthesis indicated that parents perceived T1DM as a difficult diagnosis that contributed to significant family disruption.	N/A
[22] *	*n* = 257 preadolescents (age ranged of 11 to 14 years) with T1DM and their primary caregivers (91% mothers).	Parental distress (sum of depression, anxiety and stress symptoms); parental self-efficacy; parents diabetes management	Parents reported experiencing depression symptoms. Less distress indirectly related to greater monitoring through higher parental self-efficacy and more authoritative parenting then leading to better HbA1c.	Beck Depression Inventory-Second Ed. (BDI-II): self-report assessing depression symptoms (clinical total score cut-off: 29). Pediatric Inventory for Parents (PIP): self-report assessing parents’ parenting stress specific to caring for a child with a chronic disease. It assesses both the Difficulty and Frequency of the stress-eliciting situations. Hypoglycemic Fear Survey-Parents (HFS-P): self-report assessing anxiety referred to hypoglycemia. Self-Efficacy for Diabetes Self-Management Scale-Parent (SEDSM-P): self-report assessing parents perceived self-efficacy regarding diabetes tasks-related management. Parental Monitoring Diabetes Care Scale (PMDC): self-report assessing the frequency with which parents monitor specific diabetes-related tasks. 24-h DI: interview assessing the percentage of parents’ blood glucose check. Glycemic Control: glycosylated Hemoglobin (A1c) indexes average blood glucose levels.
[23] **	*n* = 147 parents (130 mothers, 13 fathers, 2 grandmothers, and 2 aunts) of adolescents (age ranged of 13 to 18 years) with T1DM.	Parental Distress (sum of depression and anxiety symptoms); Parents diabetes management	The relationship between parents’ psychological distress and HbA1C levels were stronger for symptoms of depression compared to symptoms of anxiety.	State-Trait Anxiety Inventory (STAI-T)—Trait subscale: self-report assessing trait anxiety symptoms (clinical total score cut-off: 40). Center for Epidemiologic Studies Depression (CESD): self-report assessing depression symptoms (clinical total score cut-off: 16). Pediatric Assessment in Diabetes-Parent version (PAID-P): self-report assessing the burden related to the child diabetes management. [parental distress]. Glycemic Control: Hemoglobin A1c values measured through the DCA 2000.
[24] *	*n* = 88 dyads (92% mothers) comprising children with T1DM (56.8% girls; age ranged of 8 to 18 years), and one parent (age ranged of 29 to 59 years). *n* = 121 (94.2% mothers) dyads, comprising healthy children (55.4% girls; age ranged of 8 to 18 years) and one parent (age ranged of 30 to 59 years).	Family cohesion, parental stress, and emotional adjustment (anxiety and depression symptoms)	Parental diabetes-specific distress negatively impacts upon family cohesion and vice versa.	Family Environment Scale (FES)—Cohesion subscale: self-report assessing perceived commitment toward the family and of the family members supportiveness and helpfulness. Hospital Anxiety and Depression Scale (HADS): self-report assessing anxiety and depression symptoms aimed at the general population. Parenting Stress Index-Short Form (PSI-SF), Parental Distress subscale: self-report assessing parents distress referred to caring and rearing a child.
[25] **	*n* = 125 families of 5- to 9-years-old children with new-onset T1DM completed study measures at baseline, *n* = 102 at 6-month follow-up, and *n* = 89 at 12-month follow-up.	Depression; T1DM-specific distress concerning daily T1DM management; worries about the future and long-term complications	Most of parents experienced paediatric specific distress after their children diagnosis of T1DM, which seems to be exacerbated by parental depression symptoms. Parents with depression symptoms reported lower levels of daily T1DM-specific distress compared to parents without these symptoms at baseline. Parents who experience depression symptom at baseline showed a smaller reduction of them at follow-up.	The Problems Ares in Diabetes Survey—Parent Revised Version (PAID-PR): self-report assessing parents distress specific to the burden associated to T1DM. Center for Epidemiologic Studies Depression (CESD): self-report assessing depression symptoms (clinical total score cut-off: 16). Glycemic Control: Hemoglobin A1c indexing average blood glucose levels in the preceding three months.
[26] *	*n* = 38 parents (32 mothers, 6 fathers) of children with T1DM (age ranged of 2 to 7 years).	Parental stress; depression	Parents report ranging between mild to moderate depression symptoms. Paediatric parenting stress is common in parents of young children with T1DM.	Pediatric Inventory for Parents (PIP): self-report assessing parents’ parenting stress specific to caring for a child with a chronic disease. It assesses both the Difficulty and Frequency of the stress-eliciting situations. Beck Depression Inventory-Second Ed. (BDI-II): self-report assessing depression symptoms. Hypoglycemia Fear Survey-Parents of Young Children (HFS-PSYC): self-report adapted to measure the anxiety related to hypoglycemia in parents of young children with diabetes.
[27] *	*n* = 195 parents of children (less than 12 years old) with T1DM.	Depression; stress	Several caregivers reported elevated symptoms of depression. The best predictors of depression symptoms for caregivers: lower caregivers’ education level, higher family stress, children older age, and poorer glycaemic control.	Family Stress Scale (FSS): self-report developed to assess illness-related stress and adapted to parents with children with T1DM. Center for Epidemiologic Studies Depression (CESD): self-report assessing depression symptoms (clinical total score cut-off: 16). Glycemic Control: Hemoglobin A_1_C (HbA1C) indexing average blood glucose levels in the preceding three months. Higher levels index worst control.
[28] *	*n* = 61 parents of children and adolescents (age ranged of 10 to 17 years) with T1DM.	Depression; parents diabetes management	Parental depression symptoms indirectly effect children and adolescents’ depressive symptoms and children and adolescents’ metabolic control through parental involvement and monitoring. Higher levels of parental depression relate to lower monitoring, inconsistent discipline, and lower parental involvement/warmth diabetes management.	Brief Symptoms Inventory 18 (BSI 18)—depression subscale: self-report assessing depression symptoms (clinical cut-off: 65). Alabama Parenting Questionnaire: self-report assessing general parenting skills. Considered subscales are “poor monitoring”, “involvement” and “inconsistent discipline”. Higher scores index greater warmth. Glycemic Control: Hemoglobin A_1_C (HbA1C) indexing average blood glucose levels in the preceding three months.
[29] *	*n* = 108 mothers (age ranged of 28 to 52 years) and their children with T1DM (60% females; age ranged of 8 to 12 years)	Depression	High levels of family general stress results as one of the risk factors for the onset of depression symptoms in parents. Maternal depression negatively affects children adjustment through its influence on children’s quality of life, coping, and family functioning.	Center for Epidemiologic Studies Depression (CESD): self-report assessing depression symptoms (clinical total score cut-off: 16). Diabetes Responsability and Conflict Scale (DRCS): self-report assessing the perceived distribution of diabetes-management related responsibilities and conflict between parents and children.
[30] *	*n* = 73 parents (97% mothers) of young child (age ranged of 2 to 6 years) with T1DM.	Parental stress; anxiety; parents diabetes management	Greater general anxiety and paediatric parenting stress associates with parents’ report of more problematic child behaviour while HbA1c monitoring was not influenced.	Pediatric Inventory for Parents (PIP): self-report assessing parents’ parenting stress specific to caring for a child with a chronic disease. It assesses both the Difficulty and Frequency of the stress-eliciting situations. State-Trait Anxiety Inventory (STAI): self-report assessing state and trait anxiety symptoms (clinical total score cut-off: 1 standard deviation above the sample mean). 24-h recall interview: two interviews assessing completion of diabetes-related tasks.
[31] *	*n* = 43 mothers-children dyads (age ranged of 8 to 15 years) with T1DM.	Parental distress; parenting stress	Maternal distress variables were strongly interrelated, with depression resulting as the greatest predictor of negative outcome for the children’s diabetes compared to anxiety.	Pediatric Inventory for Parents (PIP): self-report assessing parents’ parenting stress specific to caring for a child with a chronic disease. It assesses both the Difficulty and Frequency of the stress-eliciting situations. Hospital Anxiety and Depression Scale (HADS): self-report assessing anxiety and depression symptoms aimed at the general population.
[32] *	*n* = 28 mothers-children with T1DM dyads (11 boys; 17 girls; age ranged of 8 to 19 years).	Parental stress; anxiety	Greater maternal stress associates to both internalizing and externalizing symptoms of the child with T1DM	Pediatric Inventory for Parents (PIP): self-report assessing parents’ parenting stress specific to caring for a child with a chronic disease. It assesses both the Difficulty and Frequency of the stress-eliciting situations.
[33] *	*n* = 465 (85.2% mothers) main caregivers of children and adolescents with T1DM (age ranged of 9 and 18 years).	Stress; anxiety; Depression	Caregivers of children and adolescents diagnosed with diabetes were those with the highest levels of stress compared to parents of healthy children and adolescents. Age negatively associates with anxiety symptoms.	Pediatric Inventory for Parents (PIP): self-report assessing parents’ parenting stress specific to caring for a child with a chronic disease. It assesses both the Difficulty and Frequency of the stress-eliciting situations. Hospital Anxiety and Depression Scale (HADS): self-report assessing anxiety and depression symptoms aimed at the general population.
[34] *	*n* = 12 parental couples (Mothers. mean age = 40.25, SD = 6.58; Fathers, mean age = 42.5, SD = 6.38) of children with T1DM aged of 7 to 11 years (mean age = 8.8, SD = 0.996).	Parenting stress	Mothers and fathers seem to report comparable stress levels related to parenting a child with diabetes. Mothers seem to be more preoccupied with the internal suffering of their children with T1DM as they typically show greater feelings of sadness and anxiety compared to fathers. Scarce metabolic control, mediated by fathers’ perception of their children’s behavioural problems, seem to influence a specific aspect of parenting stress related to the parent-child dyad’s dysfunctional interaction	Parenting Stress-Fourth Edition- Index-Short Form (PSI-4-SF): self-report assessing parents distress referred to caring and rearing a child.
[35] *	*n* = 24 parents (females 88%; married 92%; mean age = 34.80 years) of children with T1DM (55% males; mean age = 4.1 years).	Parental stress; anxiety; depression	Children sleep issues and behavioural insomnia associates with increased parental stress, anxiety and depression symptoms, also affecting the intense insulin regimen. No association between anxiety and depression symptoms and children’s diabetes specific issues related to sleep.	Pediatric Inventory for Parents (PIP): self-report assessing parents’ parenting stress specific to caring for a child with a chronic disease. It assesses both the Difficulty and Frequency of the stress-eliciting situations. State-Trait Anxiety Inventory (STAI): self-report assessing state and trait anxiety symptoms. Center for Epidemiologic Studies Depression (CESD): self-report assessing depression symptoms.
[36] *	*n* = 229 parents (126 mothers and 103 fathers) of adolescents with T1DM (age ranged of 12 to 18 years) and a comparison group *n* = 126 (106 mothers and 55 fathers).	Parenting Stress	Fathers of adolescents with T1DM report significantly more parenting stress compared to mothers.	Parenting Stress-Index-Short Form (PSI-SF): self-report assessing parents stress referred to caring and rearing a child.
[37] *	*n* = 134 parents (86% mothers) of children with T1DM (age ranged of 9 to 17 years).	Parental stress; self-efficacy; parents diabetes management	Parenting stress negatively relaters to children’s age and positively relates to regimen status. Perception of lower self-efficacy and greater responsibility towards diabetes management in parents.	Self-Efficacy for Diabetes scale (SED): self-report adopted to assess parent perceived capacity (self-efficacy) to manage their children diabetes. Pediatric Inventory for Parents (PIP): self-report assessing parents’ parenting stress specific to caring for a child with a chronic disease. It assesses both the Difficulty and Frequency of the stress-eliciting situations. Diabetes Family Responsability Questionnaire (DFRQ): self-report assessing parents’ level of involvement and responsibility in their children diabetes management.
[38]	*n* = 135 families (82% mothers; 16% fathers; 1% caregivers) and children with T1DM (age ranged of 10 to 16 years).	Parental self–efficacy; diabetes management	High parental self-efficacy for diabetes management and the teaching of management skills may positively influence youth’s confidence for diabetes self-management.	Maternal Self-Efficacy for Diabetes Management Scale (MSED): self-report assessing the caregiver self-efficacy regarding the daily management of diabetes-related tasks.
[39] *	*n* = 67 mothers and children with T1DM (less than 8 years old) dyads.	Anxiety; depression; parents diabetes management	21% of mothers reported clinically significant levels of anxiety symptoms. 24% of mothers reported clinically significant levels of depression symptoms. Maternal symptoms were not related to children’s metabolic control.	State-Trait Anxiety Inventory (STAI): self-report assessing state and trait anxiety symptoms (cut-off for high anxiety: 44). Center for Epidemiologic Studies Depression (CESD): self-report assessing depression symptoms (clinical cut-off: 16). Glycemic Control: Hemoglobin A_1_C (HbA1C) indexing average blood glucose levels in the preceding three months and assessed through the DCA 2000.
[40] **	*n* = 220 mothers and children with T1DM dyads (age ranged of 11 to 19 years) of which *n* = 187 dyad (control group) it was used only the utilization/charge data while for the other *n* = 118 dyad CES-D and utilization/charge data.	Depression; parents diabetes management	Maternal depression does not associate with glycaemic control; however, adolescents of mothers with high depression symptoms were twice as likely to have an emergency room visit and three times as likely to be hospitalized	Center for Epidemiologic Studies Depression (CESD): self-report assessing depression symptoms (clinical cut-off: 16). Glycemic Control: Hemoglobin A_1_C (HbA1C) indexing average blood glucose levels in the preceding 3 months.
[41] **	*n* = 82 mothers-adolescents with T1DM dyads (adolescents mean age = 12.79 years at baseline; mean age = 14.16 years at follow-up).	Depression; parents diabetes management	Parental depressive symptoms show a significant indirect effect on children depressive symptoms when the parental involvement was considered. The impact of maternal involvement varies as a function of their levels of depression symptoms and the adolescent age.	Center for Epidemiologic Studies Depression (CESD): self-report assessing depression symptoms (clinical cut-off: 16). Diabetes Responsability and Conflict Scale (DRCS): self-report assessing the perceived distribution of diabetes-management related responsibilities and conflict between parents and children. Glycemic Control: Hemoglobin A_1_C (HbA1C) indexing average blood glucose levels in the preceding 3 months. Higher levels index worse glycaemic control.
[42] *	*n* = 187 parents (82% mothers) of children and adolescents (age ranged of 10 to 18 years) with T1DM.	Depression	Caregivers with high scores on depression symptoms report high levels of youth depression symptoms at both high and low levels of youth-reported depression symptoms.	Center for Epidemiologic Studies Depression (CESD): self-report assessing depression symptoms (clinical cut-off: 16).
[43] *	*n* = 81 mothers of adolescents (age ranged of 10 to 16 years) with T1DM.	Depression; parental diabetes-distress	Most common diagnosis was depression in parents with children with T1DM. Maternal depression was the only significant predictor of adolescents’ glycaemic control.	Parent Diabetes Distress Scale (P-DDS): self-report assessing the distress referred to the self, the teen child, the relationships with the teen child and the teen child healthcare team. (moderate distress: 2–3 [mean score]; high distress: < or = 3 [mean score]). Patient Health Questionnaire (PHQ-9): self-report assessing depression symptoms as referred to DSM-V major depressive disorder. (mild depression = 5–9; moderate depression = 10–14; moderately severe depression = 15–19; severe depression = 20–27).
[44] *	*n* = 118 mothers-adolescents with T1DM (age ranged of 10 to 16 years) dyads.	Parental stress; parental distress (sum of depression anche anxiety symptoms); parents diabetes management	Mothers report diabetes-related-stress. Maternal coping is not significantly associated with adolescents’ health outcomes.	Responses to Stress Questionnaire (RSQ): self-report assessing the frequency of experiencing diabetes-related stress referred to diabetes task-related management. Center for Epidemiologic Studies Depression (CESD): self-report assessing depression symptoms (clinical cut-off: 16). State-Trait Anxiety Inventory (STAI): self-report assessing state and trait anxiety symptoms (range indicating high anxiety: 20–80). Diabetes Responsability and Conflict Scale (DRCS): self-report assessing the perceived distribution of diabetes-management related responsibilities and conflict between parents and children (range for greater parent-child conflict = 15–75). Glycemic Control: Hemoglobin A_1_C (HbA1C) indexing average blood glucose levels in the preceding 3 months and measured through the DCS 2000.
[45] *	*n* = 30 adolescents (age ranged of 10 to 16 years) with T1DM and their mothers (NA).	Diabetes-related stress; anxiety; depression; parents diabetes management	Maternal anxiety and depression symptoms were related to lower levels of child–centred parenting and higher level of hostility. Higher levels of observed parental influence (i.e., attempts to regulate or control children’s behaviour) is related to greater symptoms of depression and poorer quality of life in adolescents.	Responses to Stress Questionnaire (RSQ): self-report assessing the frequency of experiencing diabetes-related stress referred to diabetes task-related management [the items were considered to assess parent-child interaction]. Iowa Family Interaction Rating Scale (IFIRS): observational measure aimed at assessing parents’ behaviour [considered dimension: hostility; parental influence; sensitivity/child centered; positive reinforcement]. Center for Epidemiologic Studies Depression (CESD): self-report assessing depression symptoms (clinical cut-off: 16). State-Trait Anxiety Inventory (STAI): self-report assessing state and trait anxiety symptoms (cut off for high anxiety: 44). Glycemic Control: Hemoglobin A_1_C (HbA1C) indexing average blood glucose levels in the preceding 3 months and measured through the DCA 2000.
[46] *	*n* = 47 fathers and mothers of adolescents with T1DM (age ranged of 13 to 18 years).	Anxiety; parents diabetes management	Trait–anxious mothers report more responsibility referred to diabetes management tasks and perceive their adolescents as having poorer management skills. Results differ based on children age. Intervention should address maternal anxiety.	Illness Perceptions Scale-Revised: self-report assessing the distress related to diabetes. Diabetes Responsability and Conflict Scale (DRCS): self-report assessing the perceived distribution of diabetes-management related responsibilities and conflict between parents and children. State-Trait Anxiety Inventory (STAI): self-report assessing state and trait anxiety symptoms. Glycemic Control: Hemoglobin A_1_C (HbA1C) indexing average blood glucose levels in the preceding 3 months. Higher levels index worse glycaemic control.
[47] ***	*n* = 225 mothers and their young adolescents with T1DM (age ranged of 11 to 14 years)	Depression	More maternal depressive symptoms were directly related to less parental monitoring and more conflict, which in turn each were related to lower adherence	Beck Depression Inventory-Second Ed. (BDI-II): self-report assessing depression symptoms (clinical total score cut-off: 29). Parent Monitoring of Diabetes Scale (PMDS): self-report assessing the monitoring of diabetes-care related behaviours on the part of parents. 24-h recall interview: two interviews assessing completion of diabetes-related tasks.
[48] *	*n* = 43 fathers of children (age ranged of 2 to 6 years) with T1DM.	Parental stress; parents diabetes management; anxiety; self-efficacy.	Fathers’ paediatric parenting stress is positively associated with state anxiety and mother-reported difficult child behaviour. Fathers report higher self-efficacy and hope that mothers.	Pediatric Inventory for Parents (PIP): self-report assessing parents’ parenting stress specific to caring for a child with a chronic disease. It assesses both the Difficulty and Frequency of the stress-eliciting situations. 24-h recall interview: two interviews assessing completion of diabetes-related tasks. Self-Efficacy for Diabetes scale (SED): self-report adopted to assess parent perceived capacity (self-efficacy) to manage their children diabetes. Hypoglycemic Fear Survey-Parents (HFS-P): self-report assessing anxiety referred to hypoglycemia. State-Trait Anxiety Inventory-State subscale (STAI-S): self-report assessing state anxiety symptoms. Glycemic Control: Hemoglobin A_1_C (HbA1C) indexing average blood glucose levels in the preceding 3 months.
[49] *	*n* = 188 fathers and mothers of adolescents with T1DM.	Depression; anxiety; self-efficacy	Association between their the paternal perceptions of self-efficacy and the adolescent self-efficacy. Mothers’ perceptions were not reported.	Parents Self-efficacy: assessed by answering questions referred to the capacity of their teen child to manage their diabetes. State-Trait Anxiety Inventory-State subscale (STAI-T): self-report assessing trait anxiety symptoms. Center for Epidemiologic Studies Depression (CESD): self-report assessing depression symptoms (clinical cut-off: 16).
[5] **	*n* = 132 adolescents with T1DM (53% females; age ranged of 10.7 to 14.21 years) with one primary caregiver (92% mothers, 7% fathers, 1% grandmother).	Parental general stress; parent diabetes-specific stress; depression; parents diabetes management	Greater parent general stress and greater parent diabetes-specific stress were associated with poorer parent mental health. General stress predicted an increase in parental depression symptoms. Parental diabetes-specific stress was associated with more frequent HbA1c monitoring and better self-care behaviours as reported by the child.	Norris & Uhl’s chronic stress measure- financial, marital, parenting stress subscales: self-report assessing parents general stress in the past 6 months. Impact on Family Scale (IFS)-family strain, personal strain, social strain subscales: self-report measure assessing the stress referred to caring for a child with a chronic illness. Center for Epidemiologic Studies Depression (CESD): self-report assessing depression symptoms. Glycemic Control: Hemoglobin A_1C_ (HbA1C). Higher levels index worse glycemic control.

Note. T1DM = Type 1 Diabetes Mellitus; * Cross-sectional study; ** Longitudinal study; *** Experimental Case Control study; **** Systematic review; NA = N; N/A = Not Available.

## Data Availability

Not applicable.
